# Quantitative genetics of wing morphology in the parasitoid wasp *Nasonia vitripennis*: hosts increase sibling similarity

**DOI:** 10.1038/s41437-020-0318-8

**Published:** 2020-05-19

**Authors:** Shuwen Xia, Bart A. Pannebakker, Martien A. M. Groenen, Bas J. Zwaan, Piter Bijma

**Affiliations:** 1 Wageningen University & Research, Animal Breeding and Genomics, PO Box 338, 6700 AH Wageningen, The Netherlands; 2 Wageningen University & Research, Laboratory of Genetics, Droevendaalsesteeg 1, 6708 PB Wageningen, The Netherlands

**Keywords:** Evolution, Genetics

## Abstract

The central aim of evolutionary biology is to understand patterns of genetic variation between species and within populations. To quantify the genetic variation underlying intraspecific differences, estimating quantitative genetic parameters of traits is essential. In Pterygota, wing morphology is an important trait affecting flight ability. Moreover, gregarious parasitoids such as *Nasonia vitripennis* oviposit multiple eggs in the same host, and siblings thus share a common environment during their development. Here we estimate the genetic parameters of wing morphology in the outbred HVRx population of *N. vitripennis*, using a sire-dam model adapted to haplodiploids and disentangled additive genetic and host effects. The results show that the wing-size traits have low heritability (*h*^2^ ~ 0.1), while most wing-shape traits have roughly twice the heritability compared with wing-size traits. However, the estimates increased to *h*^2^ ~ 0.6 for wing-size traits when omitting the host effect from the statistical model, while no meaningful increases were observed for wing-shape traits. Overall, host effects contributed to ~50% of the variation in wing-size traits. This indicates that hosts have a large effect on wing-size traits, about fivefold more than genetics. Moreover, bivariate analyses were conducted to derive the genetic relationships among traits. Overall, we demonstrate the evolutionary potential for morphological traits in the *N. vitripennis* HVRx-outbred population, and report the host effects on wing morphology. Our findings can contribute to a further dissection of the genetics underlying wing morphology in *N. vitripennis*, with relevance for gregarious parasitoids and possibly other insects as well.

## Introduction

Winged insects, Pterygota, are often considered to be the most successful terrestrial arthropods. The ability to exploit new habitats and fast dispersal by flight have been recognized as the main causes of their ecological and evolutionary success (Mayhew 2007). Many studies have shown that wing morphology, e.g., wing size and shape, is an important determinant of aerodynamic effects on flight performance, and thus strongly influences flight behaviour and fitness (Wootton 1992; Berwaerts et al. 2002; Dudley 2002). In general, long and narrow wings give greater speed and endurance of flight, while short and wide wings give higher manoeuvrability (Norberg and Rayner 1987; Betts and Wootton 1988; Wootton 1992; Dudley 2002). Thus, depending on biological and physical environmental conditions, natural selection is expected to result in wing morphology adaptations. It is, therefore, important to understand how wing morphology can actually evolve under natural selection.

One way to study the ability of wing morphology to respond to natural selection, is to investigate the quantitative genetic components of variation in wing morphology. Phenotypic variation in morphological traits observed among individuals or between populations of the same species can result from genetic and environmental factors (Falconer and Mackay 1996; Lynch and Walsh 1998). The presence of additive genetic variance for wing morphology in natural populations is a necessary condition for a phenotypic response to natural selection. The magnitude of the additive genetic variance is commonly expressed as the narrow-sense heritability (*h*^2^), the relative fraction of the total phenotypical variation due to additive genetic variation in a population. When heritability is high, phenotypic variation is mostly due to additive (i.e., heritable) effects of genes (Falconer and Mackay 1996; Lynch and Walsh 1998). Another parameter commonly used in evolutionary studies to express the extent of additive genetic variance, is the evolvability (Houle 1992). Evolvability has been widely used to compare the evolutionary potential of natural populations, and gives an indication of the capacity of a population to respond to selection when the environment changes (Houle 1992). Evolvability in quantitative genetics is measured as the coefficient of additive genetic variation (CV_A_), which standardizes the additive genetic standard deviation by the trait mean rather than the phenotypic variation. In addition, the short-term response to natural selection depends not only on the heritabilities of the traits, but also on the genetic and phenotypic covariances among traits (Lande and Arnold 1983; Falconer and Mackay 1996; Lynch and Walsh 1998). Genetic correlations result from pleiotropy or linkage among genes controlling traits, and their values and signs measure the ability of traits to evolve independently. A non-zero genetic correlation presents a constraint (e.g., it reduces the response to multi-trait selection in the direction opposite to the genetic correlation), and it can also create a trade-off (e.g., selection for a trait may cause an unfavourable correlated response in another trait). Thus, to understand how multiple traits can evolve together, it is crucial to understand all their quantitative genetic parameters.

*Nasonia* is a genus of gregarious parasitoid wasps of blowfly pupae (Whiting 1967), and includes four species: *N. vitripennis*, *N. longicornis, N. giraulti* and *N. oneida* (Werren et al. 2010). They are often used as model species in developmental and evolutionary genetics (Werren et al. 2010). All *Nasonia* species have large wings and are capable of flight, except for *N. vitripennis* males that have small vestigial wings and are unable to fly (Weston et al. 1999; Loehlin et al. 2010a). The genetic basis of this interspecific difference in male wing size has been extensively investigated (Weston et al. 1999; Gadau et al. 2002; Loehlin et al. 2010a; Loehlin et al. 2010b), which has greatly improved our understanding of the genetic mechanisms underlying the wing size and shape differences between *Nasonia* species. However, no studies are available on quantitative genetic parameters for wing morphology within *Nasonia* species.

Compared with diploid species, relatively few studies on quantitative genetic parameters have been conducted in haplodiploids, such as *Nasonia* or other parasitoid wasps (Peire Morais 2007; Shuker et al. 2007). Similar to other Hymenoptera, *Nasonia* has a haplodiploid sex determination system (Whiting 1967), in which males develop from unfertilized eggs and are haploid, while females develop from fertilized eggs and are diploid. Thus, in haplodiploids, fathers only contribute genes to daughters, and quantitative genetic analysis of haplodiploids, such as *Nasonia*, needs to be adjusted to account for the resulting genetic relationships among individuals (Liu and Smith 2000).

Moreover, *Nasonia* is a gregarious parasitoid, which can lay up to 60 eggs in a single Dipteran pupa (Whiting 1967). This could create environmental similarity between offspring developing within the same pupa (here referred to as “host”), which needs to be accounted for in the statistical model to avoid confounding environmental with genetic effects. In addition to statistical confounding, the quality of the hosts is crucial for development and size, and also affects adult longevity and fecundity (Godfray 1994). Therefore, host quality can generate variation and influence the genetic architecture of the traits. Thus, a common environment effect (i.e., host effect) should be included in the analysis, not only to avoid the inflation of genetic parameter estimates, but also to quantify its effect on trait variation.

To quantify the potential of wing morphology in *Nasonia* to respond to (multi-trait) natural selection, we constructed a population consisting of half-sib families and estimated the quantitative genetic parameters of intraspecific variation in wing size and shape in an outbred population of *Nasonia vitripennis*. Our main objective is to partition phenotypic (co)variation in size and shape traits into genetic and non-genetic components. For this purpose, we adapted the linear mixed model known as the “animal model” (Henderson 1984; Kruuk 2004) to haplodiploids (1) to estimate the genetic parameters for wing traits, i.e., heritabilities, coefficients of additive genetic variance and genetic correlations, and (2) to quantify the host effect.

## Materials and methods

### *Nasonia* stock

We used the *N. vitripennis* HVRx-outbred population, which was established from strains collected from a single-field population in the Netherlands (van de Zande et al. 2014). To preserve genetic diversity across generations, the HVRx stock is maintained in the laboratory according to a fixed schedule, in which ~120 mated females in total are transferred to four new mass culture tubes to initiate the next generation (van de Zande et al. 2014). Per tube, 50 hosts (*Calliphora vomitoria* fly pupae) are provided for oviposition. To ensure optimal mixing of the wasps, the parasitized hosts are re-distributed over four new mass culture tubes before offspring emerge. Approximately 14 days are needed to complete a cycle at 25 °C and a 16-h light, 8-h dark scheme.

### Experimental design

We generated a family structure to allow estimation of genetic parameters using a mixed linear model and pedigree relationships among individuals (Henderson 1984). Half-sib families were created by making mating groups of one male (sire) with five virgin females (dams). To collect virgin wasps, a large number of parasitized hosts from the mass-reared outbred population were opened, and male and female pupae were collected separately, ~3 days before emergence. Following emergence, we put one male and five females in one tube and allowed them to mate for 2 days. After mating, we placed each female individually into a new tube with two host pupae, in order to split larval environments (i.e., the host) within full-sib families. After 2 days of oviposition, we removed the female and placed the hosts in separate tubes kept at 25 °C and a 16-h light, 8-h dark regime. Female offspring was enclosed after 13 or 14 days. From each host, we collected three female offspring, yielding six experimental daughters per full-sib family. In total, 1889 individuals were used in this study, including 55 sires, 265 dams and 1569 female offspring. All of the hosts used in this study were provided by a single commercial supplier in a single batch.

### Morphological trait measurements

**Fig. 1:**
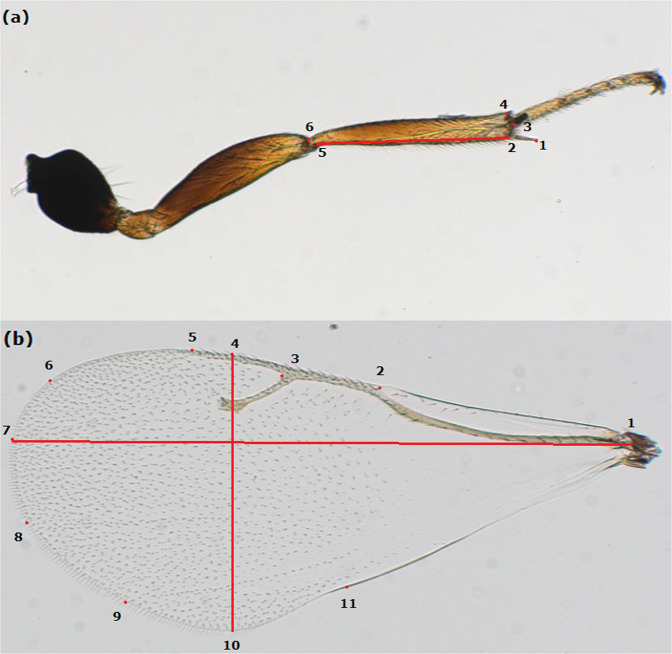
Landmarks on a Nasonia vitripennis hind tibia (**a**) and forewing (**b**), used to calculate wing morphology traits described in Table 1.

The right forewing and right hind tibia of 1569 female offspring were dissected and mounted in Euparal (Waldeck GmbH & Co. KG, Division Chroma, Münster, Germany) under coverslips on microscope slides. The right hind tibia was collected in this study, because tibia length can be used as a proxy of body size for parasitoids (Godfray 1994). We used tibia length to scale wing size, so as to remove the correlation between the wing and body size. Slides were photographed on a Zeiss Imager.A1 microscope (Zeiss AG, Göttingen, Germany) at ×2.5 magnification. Data for wing size, shape and tibia length were obtained by positioning landmarks on each digitized wing using tpsDig software (Rohlf 2013), which expresses landmarks as *x* and *y* coordinates in Cartesian space. Six landmarks were located on the spike and the joint points of the hind tibia (Fig. 1a), and 11 landmarks on the wing setae, on the wing margin and on the free ends of wing veins (Fig. 1b). To check the consistency of where we placed the landmark positions, we estimated the repeatability by re-measuring ~100 wings. A very high repeatability (~0.98) was obtained, which indicates that positioning of the landmarks is highly repeatable and consistent. The wing-size traits and tibia length were calculated from the distance between two landmarks using their coordinates (Table 1).

**Table 1:** Trait description.

Traits (units)	Description
Tibia length (µm)	Distance between the proximal and distal ends. Tibia length was measured as the distance between landmarks 2 and 5 (Fig. 1a).
Wing length (µm)	The maximum distance between the notch at the proximal edge of the costal cell and the distal part of the wing. Wing length was measured as the distance between landmarks 1 and 7 (Fig. 1b).
Wing width (µm)	The perpendicular distance between two lines parallel to the length axis. Wing width was measured as the distance between landmarks 4 and 10 (Fig. 1b).
Wing surface (µm^2^)	The area within the closed polygon defined by landmarks 1 through 11 and back to 1 (Fig. 1b).
Aspect ratio (−)	The ratio of wing length to wing width.
Scaled wing length (−)	The ratio of wing length to tibia length.
Scaled wing width (−)	The ratio of wing width to tibia length.
Wing-shape PC (−)	The first principal component of the Procrustes shape coordinate covariance matrix.

Compared with wing size, wing shape is more difficult to define, and we assessed wing-shape variation using different methods. First, wing shape was calculated as the aspect ratio, which was defined as wing length divided by wing width. Second, we investigated wing shape as scaled wing length and width in which both traits were scaled to the tibia length. In addition, we also quantified wing shape using geometric morphometrics, in which the raw coordinates digitized by tpsDig were analyzed in MorphoJ (version 1.07a, Klingenberg 2011). In MorphoJ, the Procrustes superimposition created a consensus wing shape using all 11 landmarks for all individuals. A principal component analysis (PCA) was performed using the covariance matrix of the Procrustes shape coordinates. Eigenvalues, percentages of variance explained for each PCA and the first two eigenvectors are shown in Supplementary Table S1–2 and Fig. S1. The individual first four principal components were used to assess wing-shape variation. Similar heritabilities were found for all four components. We thus only reported the results for the first component, referred to as “wing shape PC” in the following. In total, eight morphological traits were analyzed, including one body-size trait (tibia length), three wing-size traits (wing length, width and surface) and four wing-shape traits (aspect ratio, scaled wing length, width and wing-shape PC).

### Data analysis

#### Variance components

In total, records of 1569 individuals, representing 55 half-sib and 265 full-sib families, were analyzed for the above eight morphological traits. Data were analyzed with a linear mixed sire and dam model. We used a sire-dam model, rather than a full-animal model, because relationships between paternal sibs differ from those between maternal sibs in haplodiploids (see below, a fitted full-animal model using an inverted haplodiploid relationship matrix yielded identical results). In addition, host identity was included in the model, because individuals developing in the same host share the same environment, and are full siblings that may show a dominance covariance. An analysis with a permanent dam effect for all offspring of the same mother was also performed, but the dam effect was small and not statistically significant. Hence, the permanent dam effect was not included in the statistical model. Therefore, in matrix notation, our final model was

$${\mathbf{y}}\,=\,u\,+\,({\mathbf{Z}}_{\rm{s}}{\mathbf{u}}_{\rm{s}}\,+\,{\mathbf{Z}}_{\rm{d}}{\mathbf{u}}_{\rm{d}})\,+\,{\mathbf{Z}}_{\rm{c}}{\mathbf{c}}\,+\,{\mathbf{e}},$$

where **y** = the vector of observed traits, **u**_**s**_ = a vector of sire-additive genetic effects, **u**_**d**_ = a vector of dam-additive genetic effects, **c** = a vector of host effects (“common environment” effects) and **e** = a vector of residual errors. *u* was the overall mean of phenotypic records. The sire (**u**_**s**_), dam (**u**_**d**_), and host effect (**c**) were taken as normally distributed and independent random effects. **Z**_**s**_, **Z**_**d**_ and **Z**_**c**_ were known design matrices assigning observations to the level of **u**_**s**_, **u**_**d**_ and **c**, respectively.

In haplodiploids, female offspring of the same (haploid) father all share his full paternal haplotype. For this reason, the sire variance in the above model equals half of the additive genetic variance. Female offspring of the same (diploid) mother, in contrast, share only 50% of their maternal haplotype because of Mendelian segregation and recombination. Thus, as in diploids, the dam variance in the above model equals one-quarter of the additive genetic variance. Thus, the above sire-dam model is a type of reduced animal model (Quaas and Pollak 1980), but the dam variance equals half the sire variance, $\sigma _{\rm{d}}^2 = 0.5\,\sigma _{\rm{s}}^2$. We forced the dam variance to be equal to half the sire variance in our sire-dam model, and calculated the additive genetic variance as twice the sire variance, $\sigma _{\rm{a}}^2 = 2\,\sigma _{\rm{s}}^2$. Phenotypic variance equals $\sigma _{\rm{p}}^2 = \sigma _{\rm{s}}^2 + \sigma _{\rm{d}}^2 + \sigma _{\rm{c}}^2 + \sigma _{\rm{e}}^2 = 1.5\sigma _{\rm{s}}^2 + \sigma _{\rm{c}}^2 + \sigma _{\rm{e}}^2$, where $\sigma _{\rm{c}}^2$ is the variance of host effects and $\sigma _e^2$ is the residual variance. Note that Mendelian sampling variance (which is part of the residual variance) only comes from mothers, and is equal to 0.25 $\sigma _{\rm{a}}^2$ rather than the usual 0.5 $\sigma _{\rm{a}}^2$ in diploids.

All analyses were performed using the ASReml software (Gilmour et al. 2012). The genetic variance components were estimated by restricted maximum likelihood, while the effects were predicted with the best linear-unbiased prediction method.

#### Heritability and phenotypic and genetic correlations

For the estimation of heritabilities and the variance due to host effects, we used estimates of single-trait analysis. Heritabilities were calculated as

$$h^2 = \frac{{2\sigma _{\rm{s}}^2}}{{1.5\sigma _{\rm{s}}^2 + \sigma _{\rm{c}}^2 + \sigma _{\rm{e}}^2}},$$

In addition, the relative variance due to the host effects was calculated as

$${\mathrm{c}}^2 = \frac{{\sigma _{\mathrm{c}}^2}}{{1.5\sigma _{\mathrm{s}}^2 + \sigma _{\mathrm{c}}^2 + \sigma _{\mathrm{e}}^2}}.$$

The significance of variance components was tested using log-likelihood-ratio tests (LRT, Lynch and Walsh 1998)

$${\rm{LR}} = - 2\left( {{\rm{LogL}}_{\rm{R}} - {\rm{LogL}}_{\rm{F}}} \right),$$

where LogL_R_ is the log of the restricted likelihood of the reduced model and LogL_F_ is the log of the restricted likelihood of the full model. We tested variance components one at a time, using a Chi-square (*χ*^2^) distribution with one degree of freedom. When *α* = 0.05, the critical value was 3.84.

We also calculated the coefficient of additive genetic variation (CV_A_) for wing size and shape traits from the estimated genetic components as ${\rm{CV}}_{\rm{A}} = \frac{{100 \ast \sqrt {V_A} }}{{\overline X }}$, where V_A_ is the additive genetic trait variance and $\overline X$ is the trait mean.

To evaluate whether different morphological traits share a common genetic basis, we performed a multivariate analysis. As this analysis did not converge, instead, we estimated genetic correlations between traits, using a bivariate version of the linear mixed sire-dam model shown above with

$$r_{{\rm{g}}_{12}} = \frac{{\sigma _{s_{12}}}}{{\sqrt {\sigma _{s_{1}}^2\;\sigma _{s_{2}}^2} }},$$

where *σ*_s12_ is the additive genetic sire covariance between two traits (traits 1 and 2).

In addition, some wing-shape traits were defined as the ratio between two size traits (e.g., aspect ratio = wing length/width). To avoid the concern over autocorrelation, we log-transformed all traits and repeated the above analysis with transforming data. We also calculated allometry slopes to examine the patterns of allometry in these traits (Supplementary Information).

## Results

All morphological traits measured in the outbred HVRx *N. vitripennis* population exhibited variation (Table 2). The outcomes of the likelihood-ratio test for significance of variance components are presented in Table 3. All wing and tibia traits showed significant evidence of additive genetic effects (LRT: *p* < 0.05, Table 3). Apart from wing-shape PC, the estimates of heritability for wing-shape traits are about twice as large as the heritabilities for wing-size traits, around 0.10 for size traits and 0.25 for wing-shape traits. In contrast to the heritabilities, estimates of evolvability are slightly larger for wing-size traits than for wing-shape traits. Hence, when genetic variability is expressed relative to the mean trait value rather than the total phenotypic variance, wing-size traits show the most additive genetic variation. Remarkably, large host effects (*c*^2^) were found for size traits, but not for wing-shape traits (Table 3). Host effects explain more than 50% of phenotypic variance for wing-size traits, whereas host effects explain only less than 10% for wing-shape traits.

**Table 2:** Means, standard deviation (SD), coefficients of phenotypic variation (CV) and minimum and maximum values for morphology traits measured in *N. vitripennis*.

Traits (units)	Mean	SD	CV (%)	Minimum	Maximum
Tibia length (µm)	642.01	38.02	5.92	356.35	729.75
Wing length (µm)	2013.52	90.76	4.51	1634.13	2233.80
Wing width (µm)	932.11	44.98	4.83	740.55	1041.98
Wing surface (µm^2^)	1.14 × 10^6^	1.03 × 10^5^	9.08	7.39 × 10^5^	1.41 × 10^6^
Aspect ratio (−)	2.16	0.03	1.53	2.01	2.35
Scaled wing length (−)	3.13	0.08	2.67	2.83	3.45
Scaled wing width (−)	1.45	0.04	2.73	1.31	1.61
Wing-shape PC (−)	0	0.02	–	0.058	−0.063

**Table 3:** Estimated variance component effect for wing morphology traits and tibia length.

Traits	$\sigma _{\rm{a}}^2$	$\sigma _{\rm{c}}^2$	$\sigma _{\rm{p}}^2$	*h*^2^	LogL_F_	LogL_R_	CV_A_ (%)	*c*^2^
Tibia length	122.20	492.33	1435.00	0.09 ± 0.04	−6085.05	−6089.66	1.72	0.34 ± 0.03
Wing length	601.64	4553.36	8136.90	0.07 ± 0.04	−6985.99	−6988.39	1.22	0.56 ± 0.04
Wing width	214.80	1053.17	1996.80	0.11 ± 0.05	−6246.46	−6251.41	2.19	0.53 ± 0.04
Wing surface	9.32 × 10^8^	5.94 × 10^9^	1.05 × 10^10^	0.09 ± 0.04	2809.4	2806.18	2.68	0.56 ± 0.04
Aspect ratio	2.79 × 10^−4^	8.24 × 10^−5^	1.10 × 10^−3^	0.25 ± 0.05	4254.6	4218.88	0.78	0.08 ± 0.03
Scaled wing length	1.39 × 10^−3^	5.69 × 10^−4^	6.97 × 10^−3^	0.20 ± 0.04	2771.15	2742.51	1.19	0.08 ± 0.03
Scaled wing width	3.82 × 10^−4^	1.01 × 10^−4^	1.55 × 10^−3^	0.25 ± 0.05	4012.9	3969.89	1.35	0.07 ± 0.03
Wing-shape PC	2.85 × 10^−5^	1.77 × 10^−5^	3.31 × 10^−4^	0.09 ± 0.03	4937.99	4930.83	–	0.05 ± 0.03

$\sigma _a^2$ additive genetic variance, $\sigma _c^2$ variance of host effects, $\sigma _p^2$ phenotypic variance, *h*^*2*^ estimated effect of heritability with standard errors, *LogL*_*F*_ the log of the restricted likelihood of the full model, *LogL*_*R*_ the log of the restricted likelihood of the reduced model, *CV*_*A*_ coefficient of additive genetic variation and *c*^*2*^ standardized variance due to host effects.

Phenotypic and genetic correlations were consistent for all pairs of traits (Table 4). Both phenotypic and genetic correlations among wing-size traits are very high, close to 1. The high correlations suggest the existence of both genetic and non-genetic factors that are common to wing-size traits, so that individuals with, e.g., longer wings, also tend to have wider wings and a larger wing surface. Similarly, tibia length showed a high positive correlation with wing-size traits, both phenotypically and genetically. Some significant correlations were also found among wing-shape traits. For instance, the genetic and phenotypic correlations are high between scaled wing length and width, as 0.83 and 0.84, respectively. In contrast, most genetic correlations between size and wing-shape traits were not significant.

**Table 4:** Estimated genetic (above diagonal) and phenotypic (below diagonal) correlations with their standard errors (SE) in brackets.

	Tibia length	Wing length	Wing width	Wing surface	Wing aspect ratio	Scaled wing length	Scaled wing width	Wing-shape PC
Tibia length	–	0.67* (0.17)	0.59* (0.17)	0.69* (0.15)	−0.11 (0.22)	−0.68* (0.15)	−0.51* (0.17)	0.13 (0.32)
Wing length	0.77* (0.01)	–	0.86* (0.07)	0.97* (0.02)	−0.13 (0.25)	0.07 (0.26)	0.13 (0.25)	−0.03 (0.34)
Wing width	0.76* (0.01)	0.95* (0.01)	–	0.95* (0.02)	−0.62* (0.17)	0.08 (0.22)	0.36 (0.20)	−0.26 (0.26)
Wing surface	0.78* (0.01)	0.98* (0.01)	0.98* (0.01)	–	−0.33 (0.23)	0.02 (0.23)	0.16 (0.23)	−0.20 (0.28)
Aspect ratio	−0.17* (0.03)	−0.08* (0.03)	−0.39* (0.03)	−0.24* (0.03)	–	0.09 (0.16)	−0.51* (0.12)	0.24 (0.23)
Scaled wing length	−0.61* (0.02)	−0.06* (0.03)	−0.14* (0.03)	−0.11* (0.03)	0.27* (0.03)	–	0.83* (0.05)	0.22 (0.19)
Scaled wing width	−0.50* (0.02)	−0.01 (0.03)	0.08* (0.03)	0.03 (0.03)	−0.29* (0.03)	0.84* (0.01)	–	−0.07 (0.18)
Wing-shape PC	0.02 (0.03)	0.005 (0.03)	0.0006 (0.03)	0.01 (0.03)	−0.07 (0.03)	−0.002 (0.03)	0.02 (0.03)	–

*Significant at *P* < 0.05.

In addition, we found similar heritabilities and correlations for the log-transformed traits as for the original traits (supplementary Table S5–6). We therefore only show results derived from original traits in the following discussion because they have real biological meaning. We also show the allometry relationships between all traits (Supplementary Table S7). For instance, wing surface has a positive allometry with tibia length as *α* > 1, which means wing surface grows faster than tibia length. While the rest of traits grow slower than tibia length and have a negative allometry with tibia length as *α* < 1.

## Discussion

In order to understand genetic variation in wing morphology in Pterygota insects, we estimated genetic parameters for wing size and shape in an outbred population of *N. vitripennis*. Low- (~0.10) -to-moderate (~0.25) heritabilities were found for wing-size and wing-shape traits. However, evolvabilities of all of the traits measured as their CV_A_ were low, ranging from 1.19 to 2.68%. Our evolvability estimates agree with Houle (1992), who observed similar values for wing length in *Drosophila melanogaster*. The similarity between estimates of evolvability for wing morphology indicates that wing morphology traits have a low capacity to respond to selection when the response is measured relative to the trait average.

Wing-size traits generally had lower heritabilities compared with wing-shape traits (Table 3). An explanation for these lower heritabilities is the large host effect. We could disentangle additive genetic effects from host effects because (i) our data contained both full- and half-siblings, and (ii) each full-sib family emerged from two distinct hosts. The results show that the host environment (indicated by *c*^2^) had a large and highly significant effect, and explained about half of phenotypic variance for wing- and body-size traits, but not for wing-shape traits (Table 3). Wing-shape traits, including aspect ratio, scaled wing length and width, were defined as the ratio of size traits, and thus the host effects on them had been scaled out, leading to smaller phenotypic variance. Small host effects were also found for wing-shape PC, which is a size-free trait (i.e., this PC was estimated based only on coordinates in Cartesian space and not on size traits). Thus, host effects have only limited impact on wing-shape traits. In contrast to wing-shape traits, developing in the same host generated an increased similarity of wing-size traits between siblings. This finding suggests that natural selection may have only limited access to the genetic variation for these traits because most of the phenotype on which selection may act is due to the developmental host. It is therefore important to investigate the causes of this host effect, for instance, through the hosts’ nutritional composition, as well as how (genetic variation for) the mother’s host selection behaviour exerts selection on the wing size and shape phenotype of her offspring (see below).

In addition, the large host effects may also explain the low heritability estimates compared with other studies. We have found no other studies on the quantitative genetics of wing morphology in *Nasonia*. However, much higher heritability estimates of wing traits have been reported in *Drosophila* and other winged insect species (Messina 1993; Hoffmann and Schiffer 1998; Bitner-Mathe and Klaczko 1999a; Matta and Bitner-Mathe 2004; Moraes et al. 2004; Moraes and Sene 2004). For example, estimated heritabilities for female wing length and width were 0.65 and 0.58, respectively, in *Drosophila melanogaster* (Hoffmann and Schiffer 1998), while heritability estimates of the aspect ratio range from 0.30 to 0.62 (Bitner-Mathe and Klaczko 1999a; Matta and Bitner-Mathe 2004; Moraes et al. 2004). The relatively low heritabilities found in our study indicate either lower genetic variance and/or higher non-genetic variance in our population. The estimate of evolvability (CV_A_) for wing length in *Nasonia* is similar to the value found in *Drosophila melanogaster* (1.22 in *Nasonia* vs. 1.56 in *Drosophila*, Houle 1992). These similar CV_A_ estimates suggest that the level of genetic variation may not be the main reason of low heritabilities found in our study. Furthermore, the larval densities in *Drosophila* heritability studies are generally all controlled, reducing the variance introduced by larval competition. Similar to the host effect observed in our study, several studies have shown an effect of larval densities on *Drosophila* morphological traits, including wing morphology (DeMoed et al. 1997; Bitner-Mathe and Klaczko 1999b).

Interestingly, when omitting the host effect from the statistical model, the estimated heritability for wing length, for example, increased by a factor of eight (from 0.07 to 0.58). This result shows that inclusion of a host effect is essential when estimating genetic parameters for size-related traits in *Nasonia*. The effect of host quality on parasitoid size has been reported long ago (see Godfray 1994). Rivers and Denlinger (1994) also reported an effect of the host on body size in *N. vitripennis*, where body size increases with the weight of the host. It is, therefore, important to realize the contribution of hosts to the variation between individuals. To our knowledge, this is the first study to quantify the extent of host effects on the variation of quantitative traits in *N. vitripennis*. For most parasitoids, host quality affects their life-history traits and behaviour (Godfray 1994). In gregarious species (e.g., *N. vitripennis*), the effect of the host on a single offspring depends not only on host size (reflecting the total amount of food), but also on the number of parasitoids developing within the host (called “clutch size”). In *N. vitripennis*, sibling competition increases with clutch size, and has stronger negative effects on the body size of females than on males (Sykes et al. 2007). In addition, *Nasonia* females appear to be able to sense host quality and adapt their reproductive behaviour accordingly. They adjust the proportion of male offspring (sex ratio) according to host condition, laying small eggs with a large proportion of males into poor hosts (Rivers and Denlinger 1994; West and Rivero 2000; Wang et al. 2013). Conversely, traits such as host-, clutch size and sex ratio can be used as indicators of host quality (West and Rivero 2000). Unfortunately, we did not record host size, sex ratio or clutch size in this study. In future studies, host effect could be standardized by these indicators, or these indicators could be systematically varied to assess how they affect the ecology and evolution of natural populations.

In addition to variance components, we also investigated relationships within and between wing size and shape traits. We found strong phenotypic and genetic relationships between wing-size traits. We also found similar relationships for log-transformed traits. The genetic correlation estimates found here are comparable with values found in *Drosophila* species (Wilkinson et al. 1990; Loeschcke et al. 1999). These high genetic correlations indicate that wing-size traits share a similar genetic background. Moreover, a high genetic correlation was found between scaled wing length and width. This high genetic correlation indicates that wing length and width have substantial common genetic variation, even after correcting for body size (as measured by tibia length). In other words, wing-size traits are genetically closely related to each other on top of their dependence on body size. Further genetic studies (e.g., GWAS and/or QTL analysis) will be helpful to further substantiate this observation.

These high genetic correlations between wing-size traits indicate that these traits will not respond to natural selection independently. In other words, the response to selection of one wing-size trait depends on the selection of other wing-size traits. On the one hand, these correlations can accelerate the rate of adaptive evolution if they are in a favourable direction. On the other, however, these correlations can constrain adaptive evolution when the correlated response in another trait has a fitness cost (e.g., Lande 1979, 1982). For instance, when natural selection would favour long and narrow wings because they give greater speed and endurance of flight, the strong positive genetic correlation between wing length and width constrains response to selection for long and narrow wings. To quantify such constraints, we calculated pairwise conditional evolvability for traits (Hansen et al. 2003; Supplementary Table S4). We observed considerable reductions in evolvability, especially between size traits. This suggests that size traits in *Nasonia* have limited ability for adaptive response to selection when natural selection constrains the change in other size traits.

Surprisingly, we observed a weak negative phenotypic correlation between the aspect ratio and wing length. This is surprising because it means that individuals with longer wings have shorter wings when measured relative to their wing width (remember that aspect ratio is wing length/width). All eigenvalues of our genetic, host and residual covariance matrices were non-negative, meaning the estimates are statistically possible, and the negative correlation does not necessarily imply estimation error. The correlation between wing length and aspect ratio not only depends on the (co)variances of wing length and width, but also on the mean values of those traits (Van Noordwijk and De Jong 1986; Stuart and Ord 1994). Given the values presented in Tables 2–4, a relative increase in wing length goes together with an even greater relative increase in wing width, resulting in a decrease of the aspect ratio. This agrees with the negative correlation between wing length and aspect ratio. A genetic study of wing-size differences between *N. vitripennis* and *N*. *giraulti* also indicated that wing width increased more than wing length when the overall size of wings increased in an introgression line created by crossing *N. vitripennis* females and *N*. *giraulti* males (Weston et al. 1999). However, the phenotypic correlation between the two traits is very small, and the genetic correlation was not significantly different from zero, implying that selection for the aspect ratio would have a limited effect on wing length.

To conclude, we found variation for wing size and shape traits among individuals from an outbred *N. vitripennis* population. By applying an adapted version of the “animal model”, we further demonstrated that wing size and shape contained significant additive genetic variation in the *N. vitripennis* HVRx-outbred population. Remarkably and importantly, we found that hosts rather than genetics explained most of the phenotypical variation in wing-size traits. Our findings also demonstrate the importance of accounting for host effects to avoid very severe bias in the estimates of heritability. Our findings reported here increase the understanding of heritable variation for wing morphology in *Nasonia*. By combining this knowledge with the wealth of genetic tools available for *Nasonia*, it facilitates the further genetic dissection of wing morphology in *N. vitripennis* using tools such as genome-wide association and genomic prediction.

## Supplementary information

Supplementary

Supplementary Table S2

## Data Availability

The raw data underlying the paper are available from the Dryad Digital Repository: 10.5061/dryad.rxwdbrv58.

## References

[CR1] Berwaerts K, Van Dyck H, Aerts P (2002). Does flight morphology relate to flight performance? An experimental test with the butterfly *Pararge aegeria*. Funct Ecol.

[CR2] Betts CR, Wootton RJ (1988). Wing shape and flight behaviour in butterflies (lepidoptera: papilionoidea and hesperioidea): a preliminary analysis. J Exp Biol.

[CR3] Bitner-Mathe BC, Klaczko LB (1999). Heritability, phenotypic and genetic correlations of size and shape of *Drosophila mediopunctata* wings. Heredity.

[CR4] Bitner-Mathe BC, Klaczko LB (1999). Plasticity of *Drosophila melanogaster* wing morphology: effects of sex, temperature and density. Genetica.

[CR5] DeMoed GH, DeJong G, Scharloo W (1997). Environmental effects on body size variation in *Drosophila melanogaster* and its cellular basis. Genet Res.

[CR6] Dudley R (2002). Mechanisms and implications of animal flight maneuverability. Integr Comp Biol.

[CR7] Falconer DS, Mackay TFC (1996). Introduction to quantitative genetics.

[CR8] Gadau J, Page RE, Werren JH (2002). The genetic basis of the interspecific differences in wing size in *Nasonia* (Hymenoptera; Pteromalidae): Major quantitative trait loci and epistasis. Genetics.

[CR9] Gilmour AR, Gogel BJ, Cullis BR, Thompson R (2012). ASReml user guide release 4.0.

[CR10] Godfray HCJ (1994) Parasitoids behavioral and evolutionary ecology. Princeton University Press Books, Princeton

[CR11] Hansen TF, Armbruster WS, Carlson ML, Pelabon C (2003). Evolvability and genetic constraint in *Dalechampia blossoms*: Genetic correlations and conditional evolvability. J Exp Zool Part B.

[CR12] Henderson CR (1984). Applications of linear models in animal breeding.

[CR13] Hoffmann AA, Schiffer M (1998). Changes in the heritability of five morphological traits under combined environmental stresses in *Drosophila melanogaster*. Evolution.

[CR14] Houle D (1992). Comparing evolvability and variability of quantitative traits. Genetics.

[CR15] Klingenberg CP (2011). MorphoJ: an integrated software package for geometric morphometrics. Mol Ecol Resour.

[CR16] Kruuk LEB (2004). Estimating genetic parameters in natural populations using the ‘animal model’. Philos Trans R Soc Lond Ser B-Biol Sci.

[CR17] Lande R (1979). Quantitative genetic analysis of multivariate evolution, applied to brain: body size allometry. Evolution.

[CR18] Lande R (1982). A quantitative genetic theory of life history evolution. Ecology.

[CR19] Lande R, Arnold SJ (1983). The measurement of selection on correlated characters. Evolution.

[CR20] Liu FH, Smith SM (2000). Estimating quantitative genetic parameters in haplodiploid organisms. Heredity.

[CR21] Loehlin DW, Enders LS, Werren JH (2010). Evolution of sex-specific wing shape at the widerwing locus in four species of *Nasonia*. Heredity.

[CR22] Loehlin DW, Oliveira D, Edwards R, Giebel JD, Clark ME, Cattani MV (2010). Non-coding changes cause sex-specific wing size differences between closely related species of *Nasonia*. PLoS Genet.

[CR23] Loeschcke V, Bundgaard J, Barker JSF (1999). Reaction norms across and genetic parameters at different temperatures for thorax and wing size traits in *Drosophila aldrichi* and *D. buzzatii*. J Evol Biol.

[CR24] Lynch M, Walsh B (1998). Genetics and analysis of quantitative traits.

[CR25] Matta BP, Bitner-Mathe BC (2004). Genetic architecture of wing morphology in *Drosophila* simulans and an analysis of temperature effects on genetic parameter estimates. Heredity.

[CR26] Mayhew PJ (2007). Why are there so many insect species? Perspectives from fossils and phylogenies. Biol Rev.

[CR27] Messina FJ (1993). Heritability and ‘evolvability’ of fitness components in *Callosobruchus maculatus*. Heredity.

[CR28] Moraes EM, Manfrin MH, Laus AC, Rosada RS, Bomfin SC, Sene FM (2004). Wing shape heritability and morphological divergence of the sibling species *Drosophila mercatorum* and *Drosophila paranaensis*. Heredity.

[CR29] Moraes EM, Sene FM (2004). Heritability of wing morphology in a natural population of *Drosophila gouveai*. Genetica.

[CR30] Norberg UM, Rayner JMV (1987). Ecological morphology and flight in bats (Mammalia; Chiroptera): wing adaptations, flight performance, foraging strategy and echolocation. Philos Trans R Soc Lond Ser B-Biol Sci.

[CR31] Peire Morais A (2007) The role of male courtship behaviour in prezygotic isolation in *Nasonia*: Do wasps finish what bacteria started? Doctorate thesis, University of Groningen.

[CR32] Quaas RL, Pollak EJ (1980). Mixed model methodology for farm and ranch beef cattle testing programs. J Anim Sci.

[CR33] Rivers DB, Denlinger DL (1994). Redirection of metabolism in the flesh fly, Sarcophaga bullata, following envenomation by the ectoparasitoid *Nasonia vitripennis* and correlation of metabolic effects with the diapause status of the host. J Insect Physiol.

[CR34] Rohlf FJ (2013). tpsDig. digitize landmarks and outlines. Department of Ecology and Evolution, State University of New York at Stony Brook

[CR35] Shuker DM, Phillimore AJ, Burton-Chellew MN, Hodge SE, West SA (2007). The quantitative genetic basis of polyandry in the parasitoid wasp *Nasonia vitripennis*. Heredity.

[CR36] Stuart A, Ord K (1994). Kendall’s Advanced Theory of Statistics.

[CR37] Sykes EM, Innocent TM, Pen I, Shuker DM, West SA (2007). Asymmetric larval competition in the parasitoid wasp *Nasonia vitripennis*: a role in sex allocation?. Behav Ecol Sociobiol.

[CR38] van de Zande L, Ferber S, de Haan A, Beukeboom LW, van Heerwaarden J, Pannebakker BA (2014). Development of a *Nasonia vitripennis* outbred laboratory population for genetic analysis. Mol Ecol Resour.

[CR39] Van Noordwijk AJ, De Jong G (1986). Acquisition and allocation of resources: their influence on variation in life history tactics. Am Nat.

[CR40] Wang X, Wheeler D, Avery A, Rago A, Choi JH, Colbourne JK (2013). Function and evolution of DNA methylation in *Nasonia vitripennis*. PLoS Genet.

[CR41] Werren JH, Richards S, Desjardins CA, Niehuis O, Gadau J, Colbourne JK (2010). Functional and evolutionary insights from the genomes of three parasitoid *Nasonia* species. Science.

[CR42] West SA, Rivero A (2000). Using sex ratios to estimate what limits reproduction in parasitoids. Ecol Lett.

[CR43] Weston RF, Qureshi I, Werren JH (1999). Genetics of wing size differences between two *Nasonia* species. J Evol Biol.

[CR44] Whiting AR (1967). The biology of the parasitic wasp *Mormoniella vitripennis* [*Nasonia brevicornis*] (Walker). Q Rev Biol.

[CR45] Wilkinson GS, Fowler K, Partridge L (1990). Resistance of genetic correlation structure to directional selection in *Drosophila melanogaster*. Evolution.

[CR46] Wootton RJ (1992). Functional morphology of insect wings. Annu Rev Entomol.

