# Supplementary material for: Quantitative genetics of wing morphology in the parasitoid wasp Nasonia vitripennis: hosts increase sibling similarity
Source: Heredity (Edinb). 2020 May 19;125(1-2):40–9. doi: 10.1038/s41437-020-0318-8 (PMC7413264; doi:10.1038/s41437-020-0318-8)
Supplement: Supplementary file 1 — Supplementary [file 41437_2020_318_MOESM1_ESM.docx]

**Supplementary Information**

Table S1 Eigenvalues and percentages of variance explained for PCA computed from the landmarks

|  | Eigenvalue | % Variance | Cumulative % |
| --- | --- | --- | --- |
| 1 | 0.00041582 | 24.427 | 24.427 |
| 2 | 0.00030973 | 18.195 | 42.622 |
| 3 | 0.0001753 | 10.298 | 52.92 |
| 4 | 0.00015266 | 8.968 | 61.887 |
| 5 | 0.00013296 | 7.811 | 69.698 |
| 6 | 0.00010107 | 5.937 | 75.635 |
| 7 | 0.00009526 | 5.596 | 81.231 |
| 8 | 0.00007281 | 4.277 | 85.508 |
| 9 | 0.0000567 | 3.331 | 88.839 |
| 10 | 0.00005097 | 2.994 | 91.834 |
| 11 | 0.000038 | 2.232 | 94.066 |
| 12 | 0.00003106 | 1.824 | 95.89 |
| 13 | 0.0000192 | 1.128 | 97.019 |
| 14 | 0.0000131 | 0.77 | 97.788 |
| 15 | 0.00000881 | 0.518 | 98.306 |
| 16 | 0.00000647 | 0.38 | 98.686 |
| 17 | 0.00000604 | 0.355 | 99.041 |
| 18 | 0.00000443 | 0.26 | 99.301 |
| 19 | 0.0000041 | 0.241 | 99.542 |
| 20 | 0.00000308 | 0.181 | 99.723 |
| 21 | 0.00000292 | 0.172 | 99.895 |
| 22 | 0.00000179 | 0.105 | 100 |


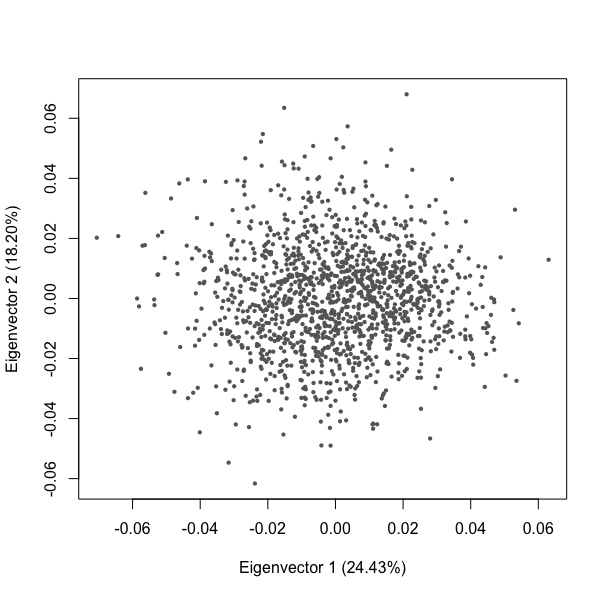


Figure S1 Plot of the first two eigenvectors of Procrustes shapes

Table S3 Estimates of (co)variance components

|  | Traits | Tibia length | Wing length | Wing width | Wing surface | Aspect ratio | Scaled wing length | Scaled wing width | Wing shape PC |
| --- | --- | --- | --- | --- | --- | --- | --- | --- | --- |
| Genetic effects | Tibia length | 61.10 |  |  |  |  |  |  |  |
|  | Wing length | 90.23 | 300.82 |  |  |  |  |  |  |
|  | Wing width | 49.88 | 151.08 | 107.40 |  |  |  |  |  |
|  | Wing surface | 1.17×10^5^ | 3.67×10^5^ | 2.15×10^5^ | 4.66×10^8^ |  |  |  |  |
|  | Aspect ratio | -0.01 | -0.03 | -0.08 | -83.51 | 1.40×10^-4^ |  |  |  |
|  | Scaled wing length | -0.14 | 0.04 | 0.02 | 9.28 | 2.81×10^-5^ | 6.94×10^-4^ |  |  |
|  | Scaled wing width | -0.06 | 0.03 | 0.05 | 48.40 | -8.40×10^-5^ | 2.94×10^-4^ | 1.91×10^-4^ |  |
|  | Wing shape PC | 3.54×10^-3^ | -1.76×10^-3^ | -0.01 | -18.42 | 9.82×10^-6^ | 2.43×10^-5^ | -3.90×10^-6^ | 1.42×10^-5^ |
| Host effects | Tibia length | 492.33 |  |  |  |  |  |  |  |
|  | Wing length | 1453.10 | 4553.36 |  |  |  |  |  |  |
|  | Wing width | 705.35 | 2148.44 | 1053.17 |  |  |  |  |  |
|  | Wing surface | 1.64×10^6^ | 5.18×10^6^ | 2.46×10^6^ | 5.94×10^9^ |  |  |  |  |
|  | Aspect ratio | -0.09 | -0.28 | -0.15 | -338.41 | 8.24×10^-5^ |  |  |  |
|  | Scaled wing length | -0.18 | -0.59 | -0.33 | -685.72 | 1.37×10^-4^ | 5.69×10^-4^ |  |  |
|  | Scaled wing width | -0.03 | -0.06 | 0.04 | -52.71 | 2.37×10^-6^ | 1.78×10^-4^ | 1.01×10^-4^ |  |
|  | Wing shape PC | -0.03 | -0.03 | 0.00 | -12.27 | -3.00×10^-5^ | 1.55×10^-5^ | 3.23×10^-5^ | 1.77×10^-5^ |
| Residuals | Tibia length | 851.01 |  |  |  |  |  |  |  |
|  | Wing length | 1042.63 | 3132.26 |  |  |  |  |  |  |
|  | Wing width | 503.20 | 1406.74 | 782.55 |  |  |  |  |  |
|  | Wing surface | 1.20×10^6^ | 3.34×10^6^ | 1.69×10^6^ | 3.90×10^9^ |  |  |  |  |
|  | Aspect ratio | -0.11 | 0.07 | -0.31 | -338.31 | 8.07×10^-4^ |  |  |  |
|  | Scaled wing length | -1.64 | 0.08 | -0.21 | -271.81 | 5.69×10^-4^ | 5.37×10^-3^ |  |  |
|  | Scaled wing width | -0.66 | -0.01 | 0.10 | 85.04 | -2.60×10^-4^ | 2.16×10^-3^ | 1.16×10^-3^ |  |
|  | Wing shape PC | 0.03 | 0.03 | 0.02 | 59.72 | -2.70×10^-5^ | -5.50×10^-5^ | -9.90×10^-6^ | 2.92×10^-5^ |

Continued table

|  | Traits | Tibia length | Wing length | Wing width | Wing surface | Aspect ratio | Scaled wing length | Scaled wing width | Wing shape PC |
| --- | --- | --- | --- | --- | --- | --- | --- | --- | --- |
| Phenotypic | Tibia length | 1435.00 |  |  |  |  |  |  |  |
|  | Wing length | 2631.08 | 8136.85 |  |  |  |  |  |  |
|  | Wing width | 1283.36 | 3781.79 | 1996.82 |  |  |  |  |  |
|  | Wing surface | 3.02×10^6^ | 9.07×10^6^ | 4.47×10^6^ | 1.05×10^10^ |  |  |  |  |
|  | Aspect ratio | -0.22 | -0.25 | -0.57 | -801.98 | 1.10×10^-3^ |  |  |  |
|  | Scaled wing length | -2.03 | -0.46 | -0.51 | -943.61 | 7.48×10^-4^ | 6.98×10^-3^ |  |  |
|  | Scaled wing width | -0.78 | -0.03 | 0.21 | 104.94 | -3.82×10^-4^ | 2.78×10^-3^ | 1.55×10^-3^ |  |
|  | Wing shape PC | 0.01 | -0.01 | 5.06×10^-4^ | 19.80 | -4.20×10^-5^ | -4.00×10^-7^ | 1.60×10^-5^ | 3.31×10^-4^ |

Conditional evolvability calculation

Conditional evolvability is a parameter to measure multivariate constraints on evolution (Hansen *et al*. 2003). Conditional evolvability measures the evolvability of a trait (trait Y) when the correlated trait (trait X) is under strong stabilizing selection. and it is determined by its conditional genetic variance (Hansen *et al*. 2003). Therefore. we computed conditional genetic variance and conditional evolvability using the (co)variance components derived from bivariate analyses (supplementary Table S2).

Conditional genetic variance (CGV) of the constrained trait Y on trait X was calculated as $CGV=\mathrm{Var}\left( Y \right)-\frac{{cov(X.Y)}^{2}}{Var(X)}$, where $\mathrm{Var}\left( X \right)$ and $\mathrm{Var}\left( Y \right)$ are the additive genetic sire variance of trait X and Y, respectively, and $cov(X.Y)$is the genetic sire covariance between two traits. We computed conditional evolvability (CE) of trait Y as $CE=\frac{\sqrt{CGV}}{\bar{Y}}$. where $\bar{Y}$ is the mean of trait Y.

Table S4 Estimated conditional genetic variance with conditional evolvability in the brackets

| Traits X  Traits Y | Tibia length | Wing length | Wing width | Wing surface | Aspect ratio | Scaled wing length | Scaled wing width | Wing shape PC |
| --- | --- | --- | --- | --- | --- | --- | --- | --- |
| Tibia length | - | 34.04 (0.91) | 37.94 (0.96) | 31.77 (0.88) | 60.29 (1.21) | 32.83 (0.89) | 43.91 (1.03) | 60.22 (1.21) |
| Wing length | 167.57 (0.64) | - | 88.31 (0.47) | 11.24 (0.17) | 295.48 (0.85) | 298.81 (0.86) | 295.85 (0.85) | 300.60 (0.86) |
| Wing width | 66.69 (0.88) | 31.539 (0.60) | - | 8.54 (0.31) | 66.76 (0.88) | 106.73 (1.11) | 93.10 (1.04) | 98.07 (1.06) |
| Wing surface | 2.42×10^5^ (1.37) | 1.74×10^5^ (0.37) | 3.70×10^7^ (0.53) | - | 4.16×10^8^(1.79) | 4.66×10^8^ (1.89) | 4.54×10^8^ (1.87) | 4.42×10^8^ (1.84) |
| Aspect ratio | 1.38×10^-4^ (0.54) | 1.37×10^-4^ (0.54) | 8.67×10^-5^ (0.43) | 1.25×10^-4^ (0.57) | - | 1.38×10^-4^ (0.54) | 1.03×10^-4^ (0.47) | 1.33×10^-4^ (0.53) |
| Scaled wing length | 3.73×10^-4^ (0.62) | 6.89×10^-4^ (0.85) | 6.89×10^-4^ (0.84) | 6.93×10^-4^ (0.84) | 6.88×10^-4^ (0.84) | - | 2.40×10^-4^ (0.50) | 6.52×10^-4^ (0.82) |
| Scaled wing width | 1.37×10^-4^ (0.81) | 1.88×10^-4^ (0.01) | 1.65×10^-4^ (0.01) | 1.86×10^-4^ (0.01) | 1.41×10^-4^ (0.01) | 6.61×10^-5^ (0.004) | - | 1.90×10^-4^ (0.01) |
| Wing shape PC | 1.40×10^-5^ (-) | 1.42×10^-5^ (-) | 1.30×10^-5^ (-) | 1.35×10^-5^ (-) | 1.35×10^-5^ (-) | 1.34×10^-5^ (-) | 1.42×10^-5^ (-) | - |

Note: Conditional evolvabilities are given as percentages in the table

References

Hansen TF. Armbruster WS. Carlson ML. Pelabon C (2003). Evolvability and genetic constraint in Dalechampia blossoms: Genetic correlations and conditional evolvability. *J Exp Zool Part B* **296B**(1)**:** 23-39.

Table S5 Estimated heritabilities (diagonal), genetic correlation (above diagonal) and phenotypic correlation (below diagonal) with their standard errors (SE) in brackets for log-transformed traits

|  | Tibia length | Wing length | Wing width | Wing surface | Wing aspect ratio | Scaled wing length | Scaled wing width | Wing shape PC |
| --- | --- | --- | --- | --- | --- | --- | --- | --- |
| Tibia length | 0.08 (0.03) | 0.67 (0.18) | 0.60 (0.18) | 0.69 (0.16) | -0.12 (0.23) | -0.69 (0.15) | -0.52 (0.18) | - |
| Wing length | 0.75 (0.01) | 0.07 (0.04) | 0.87 (0.07) | 0.96 (0.02) | -0.14 (0.26) | 0.08 (0.26) | 0.13 (0.25) | - |
| Wing width | 0.74 (0.01) | 0.95 (0.003) | 0.11 (0.04) | 0.95 (0.02) | -0.62 (0.17) | 0.07 (0.23) | 0.36 (0.20) | - |
| Wing surface | 0.76 (0.01) | 0.98 (0.001) | 0.98 (0.001) | 0.08 (0.04) | -0.34 (0.23) | 0.01 (0.25) | 0.17 (0.23) | - |
| Aspect ratio | -0.17 (0.03) | -0.09 (0.03) | -0.38 (0.03) | -0.24 (0.03) | 0.25 (0.05) | 0.09 (0.16) | -0.51 (0.12) | - |
| Scaled wing length | -0.62 (0.02) | -0.06 (0.03) | -0.14 (0.03) | -0.11 (0.03) | 0.27 (0.03) | 0.20 (0.04) | 0.83 (0.05) | - |
| Scaled wing width | -0.51 (0.02) | -0.008 (0.03) | 0.08 (0.03) | 0.02 (0.03) | -0.29 (0.03) | 0.84 (0.009) | 0.25 (0.04) | - |
| Wing shape PC | - | - | - | - | - | - | - | - |

Table S6 (co)variance components for ratio trait AR=WL/WW as an example

|  | Genetic effects | Host effects | Residuals |
| --- | --- | --- | --- |
| Var(Log(WL)) | 1.43×10^-5^ | 2.19×10^-4^ | 1.56×10^-4^ |
| Var(Log(WW)) | 2.43×10^-5^ | 2.38×10^-4^ | 1.82×10^-4^ |
| Cov(Log(WL), Log(WW)) | 1.58×10^-5^ | 2.24×10^-4^ | 1.52×10^-4^ |
| Var(Log(WL)-Log(WW))* | 0.70×10^-5^ | 0.09×10^-4^ | 0.34×10^-4^ |
| Var(Log(AR))** | 0.56×10^-5^ | 0.03×10^-4^ | 0.33×10^-4^ |

AR: wing aspect ratio, WL: wing length, and WW: wing width

*: Var(Log(WL/WW))=Var(Log(WL)-Log(WW)) derived from variance components of WL and WW

**: calculated AR as WL/WW, and log transformed AR

Regression and allometry slope calculation

We first conducted a linear regression of trait Y on X，as $Y=bX+e$. The slope b was calculated as b =$\frac{cov(X,Y)}{Var(X)}$, where $\mathrm{Var}\left( X \right)$ is the phenotypic variance of trait X, and $cov(X,Y)$ is the phenotypic covariance between the two traits. We also calculated the allometry relationship between traits X and Y, $Y=cX^{\alpha}$, or equivalently in a logarithmic form: $\log\left( Y \right)=\log\left( c \right)+\alpha log(X)$, where intercept c and allometry slope $\alpha$are constants. Similar to the above linear regression, the allometry slope of trait Y on trait X was calculated as $\alpha=\frac{\mathrm{cov}\left[ \log\left( X \right),log(Y) \right]}{\mathrm{Var}\left[ log(X) \right]}$, where $\mathrm{Var}\left[ log(X) \right]$ is the phenotypic variance of log-transformed X, and $\mathrm{cov}\left[ \log\left( X \right),log(Y) \right]$ is the phenotypic covariance between log-transformed X and Y. The estimated slope b and allometry slope $\alpha$ were computed from the (co)variance components, and are shown in Table S5.

Table S7 Estimated linear regression slope ($b$) and allometry slope ($\alpha$) of trait Y on X

|  | Traits X  Traits Y | Tibia length | Wing length | Wing width | Wing surface | Aspect ratio | Scaled wing length | Scaled wing width | Wing shape PC |
| --- | --- | --- | --- | --- | --- | --- | --- | --- | --- |
| $b$ | Tibia length | - | 0.32 | 0.64 | 2.87×10^-4^ | -199.63 | -291.51 | -503.95 | 42.13 |
|  | Wing length | 1.83 | - | 1.89 | 8.61×10^-4^ | -222.55 | -66.16 | -19.22 | -27.06 |
|  | Wing width | 0.89 | 0.46 | - | 4.24×10^-4^ | -520.88 | -72.73 | 139.03 | 1.53 |
|  | Wing surface | 2104 | 1115 | 2237 | - | -729736 | -135285 | 67900 | 59840 |
|  | Aspect ratio | -1.53×10^-4^ | -3.01×10^-5^ | -2.87×10^-4^ | -7.61×10^-8^ | - | 0.11 | -0.25 | -0.13 |
|  | Scaled wing length | -1.41×10^-3^ | -5.67×10^-5^ | -2.54×10^-4^ | -8.95×10^-8^ | 0.68 | - | 1.80 | -0.01 |
|  | Scaled wing width | -5.43×10^-4^ | -3.65×10^-6^ | 1.08×10^-4^ | 9.96×10^-9^ | -0.34 | 0.40 | - | 0.05 |
|  | Wing shape PC | 9.72×10^-6^ | -1.10×10^-6^ | 2.53×10^-7^ | 1.88×10^-9^ | -0.04 | -5.73×10^-4^ | 0.01 | - |
| $\alpha$ | Tibia length | - | 1.02 | 0.94 | 0.5 | -0.68 | -1.55 | -1.24 | - |
|  | Wing length | 0.55 | - | 0.88 | 0.47 | -0.26 | -0.11 | -0.01 | - |
|  | Wing width | 0.58 | 1.01 | - | 0.51 | -1.23 | -0.25 | 0.14 | - |
|  | Wing surface | 1.15 | 2.03 | 1.88 | - | -1.47 | -0.4 | 0.08 | - |
|  | Aspect ratio | -0.04 | -0.03 | -0.12 | -0.04 | - | 0.15 | -0.16 | - |
|  | Scaled wing length | -0.28 | -0.04 | -0.07 | -0.03 | 0.47 | - | 0.83 | - |
|  | Scaled wing width | -0.23 | -0.005 | 0.04 | 0.007 | -0.52 | 0.87 | - | - |
|  | Wing shape PC | - | - | - | - | - | - | - | - |
